# Treatment of Spleen-Deficiency Syndrome With Atractyloside A From Bran-Processed *Atractylodes lancea* by Protection of the Intestinal Mucosal Barrier

**DOI:** 10.3389/fphar.2020.583160

**Published:** 2020-11-20

**Authors:** Jiyuan Tu, Ying Xie, Kang Xu, Linghang Qu, Xiong Lin, Chang Ke, Desen Yang, Guosheng Cao, Zhongshi Zhou, Yanju Liu

**Affiliations:** ^1^ School of Pharmacy, Hubei University of Chinese Medicine, Wuhan, China; ^2^ Hubei Research Center of Chinese Materia Medica Processing Engineering and Technology, Hubei University of Chinese Medicine, Wuhan, China

**Keywords:** spleen-deficiency syndrome, Atractyloside A, network pharmacology, intestinal mucosal barrier, p38 MAPK

## Abstract

*Atractylodes lancea* (Thunb.) DC. (AL) is used in traditional Chinese medicine for the treatment of spleen-deficiency syndrome (SDS). Bran-processed *Atractylodes lancea* (BAL) has been found to be more effective than unprocessed AL. However, the compound in BAL active against SDS remains unclear. The pharmacological efficacy of BAL and its mechanism of action against SDS were investigated by HPLC-ELSD. Candidate compound AA (atractyloside A) in AL and BAL extracts was identified by HPLC-MS analysis. AA was tested in a rat model of SDS in which body weight, gastric residual rate, and intestinal propulsion were measured, and motilin (MTL), gastrin (GAS), and c-Kit were quantified by enzyme-linked immunosorbent assay. Potential targets and associated pathways were identified based on network pharmacology analysis. mRNA expression levels were measured by qRT-PCR and protein expression levels were measured by Western blot analysis and immunohistochemistry. AA increased body weight, intestinal propulsion, MTL, GAS, and c-Kit levels, while decreasing gastric residual volume and intestinal tissue damage, as same as Epidermal Growth Factor Receptor and Proliferating Cell Nuclear Antigen levels. Seventy-one potential pharmacologic targets were identified. Analysis of protein interaction, Gene Ontology (GO) functional analysis, pathway enrichment analysis, and docking and molecular interactions highlighted MAPK signaling as the potential signal transduction pathway. Validation experiments indicated that treatment with AA increased MTL, GAS, ZO-1, and OCLN levels, while reducing AQP1, AQP3, and FGF2 levels. In addition, phosphorylation of p38 and myosin light-chain kinase (MLCK) expression were inhibited. AA improved gastrointestinal function by protecting the intestinal mucosal barrier via inhibition of the p38 MAPK pathway. The results have clinical implications for the therapy of SDS.

## Introduction

Spleen-deficiency syndrome (SDS) is a common digestive disorder described in traditional Chinese medicine (TCM) texts (Wu et al., 2011). According to TCM theory, the word spleen is not synonymous anatomically, physiologically, or pathologically with the organ understood in modern medicine (Wu, 1998). Instead, spleen-deficiency syndrome refers to multisystem and multiorgan functional impairment caused by a disturbance of the digestive tract, including damage to the intestinal mucosal barrier and dysfunction of gastrointestinal motility triggered by the immune system (Li et al., 2003; Wang et al., 2015; Shi et al., 2019).

According to TCM, treatment strategies for SDS involve supplementation of Qi and strengthening the spleen (Ji et al., 2019; Ma et al., 2019a), for which *Atractylodes lancea* (AL) and its bran-processed products are used. AL is widely distributed in China and is frequently also used in the treatment of rheumatic diseases, night blindness, and influenza (Kan et al., 2011; Yu et al., 2019). In TCM, appropriate processing of herbs can either enhance clinical efficacy or reduce toxicity and side effects (Chen et al., 2018b). Stir frying with bran is a routine processing method in TCM, and the bran processing of *Atractylodes lancea* (BAL) enhances its spleen-strengthening effects. In fact, this is the sole processed product listed in the 2015 Chinese Pharmacopoeia for this syndrome.

Modern medical research has demonstrated that BAL is the most effective treatment of SDS, consistent with TCM theory (Yu et al., 2015; Xue et al., 2018; Ma et al., 2019b; Zhang et al., 2020). For example, Zhang *et al.* found that, compared with a model group, gastrointestinal hormone levels in the plasma of AL and BAL-treated animals increased, with BAL found to be more effective than AL. Ma *et al.* found that the composition of the intestinal microbiota in SDS rats was significantly different from that of healthy rats and tended to recover to normal levels after treatment with BAL. In this respect, BAL was superior to AL in terms of improvement in gastrointestinal tract function. Xue *et al.* found that the efficacy of BAL and AL could be partially attributed to digestive enzyme activity, gastrointestinal hormone levels, membrane protein activity, and changes in mitochondrial activity. BAL was more effective in treating SDS. SDS is usually accompanied by ulcers. Yu *et al.* found that BAL was more satisfactory for treating gastric ulcers than AL due to its anti-inflammatory properties and gastrointestinal-protective effects. In a previous study, we showed that an extract of AL protected the integrity of the intestinal mucosal barrier in rats with induced spleen-deficiency syndrome via the p38 mitogen-activated protein kinase (p38 MAPK) signaling pathway, through increased aquaporin protein and tight junction protein (ZO-1 and occludin) expression, and by inhibition of p38 MAPK phosphorylation (Shi et al., 2019). However, the pharmacologically active compounds in AL that improved intestinal mucosal barrier function were not identified. The intestinal mucosal barrier is a single contiguous layer of cells lining the gastrointestinal tract which plays a critical role in preventing access to harmful agents, such as endotoxins, microorganisms, and hydrolytic enzymes (Salvo Romero et al., 2015; Xue et al., 2017). Previous studies have shown that intestinal mucosal barrier function depends on the normal activity of tight junction proteins (Berkes et al., 2003; Boirivant and Strober, 2007; Plaza-Diaz et al., 2019; Xiao et al., 2019). Occludin and myosin light-chain kinase (MLCK) are major transmembrane proteins that fulfill this role. Downregulation of occludin and upregulation of MLCK enables maintenance of mucosal barrier function (Feng et al., 2016; Hou et al., 2017; Yang et al., 2019). In addition, a number of recent studies have shown that p38 MAPK signaling is involved in the regulation of tight junction protein expression, as inhibition of p38-MAPK signaling was able to protect intestinal mucosal barrier function (Li et al., 2017; Jiang et al., 2019). Based on the result of these studies, our goal was to investigate the active ingredient of AL and BAL and its pharmacological effects on SDS.

To clarify the pharmacodynamic differences between nonprocessed and bran-processed AL, systematic solvent separation of the components was performed with analysis by HPLC-ELSD to isolate and characterize the active compound, AA. Furthermore, its protective gastric effects were explored in a rat model of SDS, as little information is available about the pharmacodynamics of AA. Network pharmacology methods were then used to identify the interactions between AA and its potential targets. Initial analysis indicated that AA principally modulates the MAPK signaling cascade. Based on the results of network pharmacology, we selected a rat model of SDS to investigate the mechanisms by which AA improved intestinal mucosal barrier function. The present study focused on AA as a representative compound and demonstrated how processing AL with bran can alter its pharmacological and constituent properties. The results provide a more detailed explanation of the pharmacological effects of AA on intestinal mucosal barrier function related to the spleen-deficiency syndrome.

## Materials and Methods

### Materials and Reagents

Specimens were collected from Hubei Province in China and identified as the rhizomes of *Atractylodes lancea* (Thunb.) DC. by Jiachun Chen from Huazhong University of Science and Technology and registered as voucher specimen no. 041005. AL was stir fried with wheat bran, in accordance with the procedures described in the National Commission of Chinese Pharmacopoeia (2015). Domperidone (Dom) supplementary tablets were obtained from Xian Janssen Pharmaceutical Ltd. (Xian, China; Drug approval number: H10910003). Silica gel for use in the column was provided by Qingdao Haiyang Chemical Co., Ltd. (Qingdao, China). Chemical reagents were purchased from Sigma Chemicals Ltd. (St. Louis, MO, United States) or from the following suppliers: atractyloside A (PUSH Biotechnology, Chengdu, China), domperidone (Janssen, Xi’an, China), and hematoxylin and eosin staining solutions (Solarbio, Beijing, China). The following antibodies were supplied by Abcam (US): goat anti-rabbit IgG (ab205718), anti-occludin (ab222691), anti-p38 (ab197348), anti-p-p38 (ab4822), and anti-myosin light-chain kinase (MLCK) (ab236299).

### Identification of the Active Compound (AA) by Comparison of Crude and Bran-Processed *Atractylodes lancea*

The air-dried and powdered rhizomes of AL (10 kg) and BAL (10 kg) were extracted using an essential oil extractor and by reflux with distilled water (80 L), 3 times for 3 h each, to obtain aqueous decoctions of AL and BAL. Both extracts were then concentrated under vacuum, resuspended in water, and partitioned using n-butanol. Furthermore, the n-butanol extracts were applied to a D101 (20–60 mesh) large-aperture absorptive resin and eluted with H_2_O-EtOH (100 : 0, 50 : 50, and 5 : 95) to obtain H_2_O-EtOH (50 : 50) fractions. The alcohol was recovered, and the AL and BAL condensed to 100.0 mg/ml for analysis by high-performance liquid chromatography (HPLC) coupled to an evaporative light-scattering detector (ELSD, Agilent, United States) to compare the chemical profiles of AL and BAL and identify the active spleen-strengthening ingredient. HPLC-ELSD analysis was performed using an Agilent-1260 system coupled to a Welch HPLC C18 column (4.6 mm × 250 mm, 5 μm) maintained at 30 °C (injection volume, 20 μL). The mobile phases were aqueous phosphoric acid (A, 0.1%, v/v) and acetonitrile (B), using the following elution gradient, was performed as follows: 2% B from 0 to 10 min, 2–5% B over 10–15 min, 5–15% B over 15–45 min, 15–25% B over 45–55 min, 25–90% B over 55–70 min, and 90% B at 80 min. A preliminary pharmacodynamics comparison was performed of the three-step extractions of AL and BAL described above.

### Experimental Models and Drug Administration

Two-hundred Sprague Dawley (SD) rats (of either sex), each weighing 195–200 g, were obtained from the Center for Disease Prevention and Control in Hubei Province, China (Reg. no. SCXF (Hubei) 2008-0005). All animals were exposed to a 12 h light/dark cycle (7 am–7 pm) and had *ad libitum* access to water, in accordance with the National Institutes of Health Guide for the Care and Use of Laboratory Animals. All animal studies were approved by the Ethics Committee for Animal Research of the Affiliated Hospital of Hubei University of TCM. The experimental design was in strict accordance with the principles and guidelines recommended by the Chinese Association for Laboratory Animal Sciences (CALAS) and was approved by the Animal Ethics Committee of Hubei University of Traditional Chinese Medicine (Approval number: 00273280).

The prescribed dose of the AL is 9 g per 60 kg adult per day, as stated in the Chinese Pharmacopoeia (2015 edition), and based on the concentration of AA in AL and the conversion ratio between dosages for humans and rats, the standard dose of AA given to rats was 2.5 mg/kg. Low and high doses of 1.25 and 5 mg/kg, respectively, were also administered. The rats were randomly divided into four groups: control, model, AA (1.25, 2.5, or 5 mg/kg), and Dom (5 mg/kg). SDS was induced for 15 days in all groups other than the control group. Rats in the control and model groups received the same volume of distilled water orally.

The rat model of SDS was established in accordance with the previously published study (Liu et al., 2012), using a compound factor method, involving the administration of an irregular diet, excessive fatigue, and a humid environment. Firstly, rats were forced to swim for 15 min in a bucket filled with 25°C water to a depth of 25 cm at a regular time each day. After swimming, lard (25 ml/kg body weight) was administered with oral gavage on days 1, 3, 5, 7, 9, 11, 13, and 15 and 30% honey on days 2, 4, 6, 8, 10, 12, and 14, within a controlled temperature of 23° ± 2°C and humidity of 50–70%. The model rats were placed in cages containing 150 g of bedding material and 600 ml of water and fed in cages covered with plastic film. The normal group was fed in a normal environment. From the 8th day onwards, the model rats were moved to a normal environment for feeding. Following the induction of SDS, the appropriate drug was administered at the selected dose for 7 days, depending on grouping. During the experiments, food intake of each group was measured on days 1, 3, 5, 7, 9, 11, 13, 15, 17, 19, and 21. Average daily food intake (g/day/rat) was calculated and converted to kJ/day/rat according to the calorie content of the diet.

On the last day of the experiment, blood was collected from each rat, which was allowed to settle for 2 h at room temperature and then centrifuged at 3,000 rpm for 15 min. The clear serum supernatant, used for ELISA assays, was stored at −80°C until required. The stomach and intestine were removed and then washed with ice-cold saline. The organs were divided in half: one part was fixed in 4% paraformaldehyde for immunohistochemical examination and pathological analysis, and the other was retained and dissolved in Trizol reagent for RNA and Western blot assays. The animal experiments were reviewed and approved by the institutional ethical review committee of Hubei University of Traditional Chinese Medicine and performed in compliance with the guidelines of the Declaration of Helsinki.

### Measurement of Residual Gastric and Intestinal Propulsion Rates

After receiving drug or water for 7 days, the rats were fasted for 24 h and then were administered with a semisolid paste (0.5 ml/kg). Thirty minutes later, the rats were sacrificed with 20% urethane (10 ml/kg), and the volume of semisolid paste in the stomach and the rate of semisolid paste propulsion in the small intestine were measured. The gastric residual and intestinal propulsion rates were calculated after 30 min in accordance with the following equations: gastric residual rate (%) = (total gastric quantity-remaining gastric quantity)/semisolid paste quantity; intestinal propulsion rate (%) = distance of advance of black semisolid paste/total length of the small intestine × 100%. During the experiment, Δbody weight of the rats was measured at preselected time points in accordance with the following equations: Δbody weight (g) = final weight (g) − initial weight (g).

### Intestinal Pathology

Tissues were cut into 0.5 cm × 0.5 cm pieces and fixed in 4% paraformaldehyde. After fixation, samples were dehydrated in alcohol immersed in xylene, embedded in paraffin, and then sliced into 0.5 μm sections for HE staining and observation using a light microscope (BX51, Olympus, Japan). Slides were assessed by an experienced histologist blinded to the experimental groupings. Histological scoring was performed in a blinded fashion by a pathologist, using a combined score for inflammatory cell infiltration and tissue damage (Araki et al., 2005).

### Measurement of Serum Gastrin and Motilin Levels

Blood was collected from the abdominal aorta of the rats following anesthesia, from which serum was separated, frozen, and then thawed at 4°C when required for analysis. The serum levels of motilin and gastrin were measured using double-antibody sandwich ELISA kits, in accordance with the manufacturer’s protocol. An ELISA reader was used to measure the absorbance at 450 nm.

### Immunohistochemical Staining of C-Kit in Intestinal Tissue and PCNA and EGFR in Gastric Tissue

Tissue samples were sliced into 5 μm sections, embedded in paraffin, dewaxed with xylene, hydrated, and then stained using the SABC method, in accordance with the kit instructions. Images were analyzed semiquantitatively using an Olympus microscopic analysis system to determine optical density values.

### Quantitative Real-Time PCR

Total RNA was isolated from intestinal tissue using Trizol reagent, in accordance with the manufacturer’s instructions and then stored at −80°C until required for analysis. This procedure was conducted in RNase-free conditions. Total RNA was reverse transcribed to cDNA using a PrimeScript RT reagent kit with gDNA Eraser, in accordance with the instruction manual. Primer sequences are displayed in Supplementary Table S1. To verify the specificity of the PCR products, melt curve analysis was performed. Gene amplification was detected using a SYBR-based quantitative PCR method. The details for PCR cycling were as follows: 95°C for 1 min, followed by 40 cycles of 95°C for 20 s and 60°C for 45 s and then 95°C for 1 min. Quantification was achieved using the comparative cycle threshold (Ct) method, after normalization with the model group. Gene expression was normalized using β-actin as the internal control.

### Western Blot Analysis

Western blots were performed in accordance with standard protocols. Briefly, tissue homogenates were prepared with lysis buffer containing 1 nM PMSF. Total proteins were separated using SDS-PAGE and then transferred to polyvinylidene difluoride (PVDF) membranes (Hercules, CA, United States). The membranes were blocked for 2 h, incubated with primary antibody (1: 500) (Danvers, MA, United States) overnight, and then incubated with the secondary antibody (1 : 2000) (Cambridge, MA, United States) for 2 h at room temperature. Protein bands were detected using an ECL chemiluminescence detection kit (Li et al., 2019). The quantity of each protein was estimated by reference to a β-actin standard.

### Network Pharmacology Analysis

The structure of AA was uploaded to the Swiss Target Prediction website (Daina et al., 2019) (http://swisstargetprediction.ch/) to obtain the most probable macromolecular targets (Supplementary Table S3). The String database (https://string-db.org/) (Szklarczyk et al., 2019) was used to identify possible protein-protein interactions, followed by Gene Ontology (GO) enrichment and KEGG pathway analysis. The relationships between potential targets and target pathways were visualized using Cytoscape 2.8.3 software. GO functional enrichment analysis results were illustrated using GraphPad Prism 7 software.

### Computational Validation of AA-Target Interaction

Interactions between AA and its protein target were established and its binding modes were explored using AutoDock Tools 1.5.6 (Morris et al., 2009). The X-ray crystal structures of p38 were obtained from the RCSB Protein Data Bank (PDB) (www.rcsb.org). PyMol software (version 2.3; https://pymol.org/) was used to visualize the binding interactions between AA and p38 as a 3D model.

### Surface Plasmon Resonance Experiments

SPR experiments were performed using a Nicoya Lifesciences OpenSPR system equipped with a COOH chip. Recombinant human MAPK14 protein (2 μg/μL, AtaGennix) in 10 mM sodium acetate buffer, pH 4.5, was immobilized on a COOH sensor chip following the manufacturer’s protocol. The interaction of AA (0, 50, 200, 400, and 800 μM) was measured using PBS buffer as running buffer at a flow rate of 20 μL/min at 25°C. Data then were fit to a 1 : 1 interaction model using the analysis software TraceDrawer.

### Statistical Analysis

All statistical calculations were performed using SPSS v21.0 software. Results are expressed as means ± standard deviation. Statistical analysis was carried out by Student’s *t*-test and ANOVA test. *p* < 0.05 was considered statistically significant.

## Results

### Identification of AA as the Active Ingredient in Crude and Bran-Processed *Atractylodes lancea*

**FIGURE 1:**
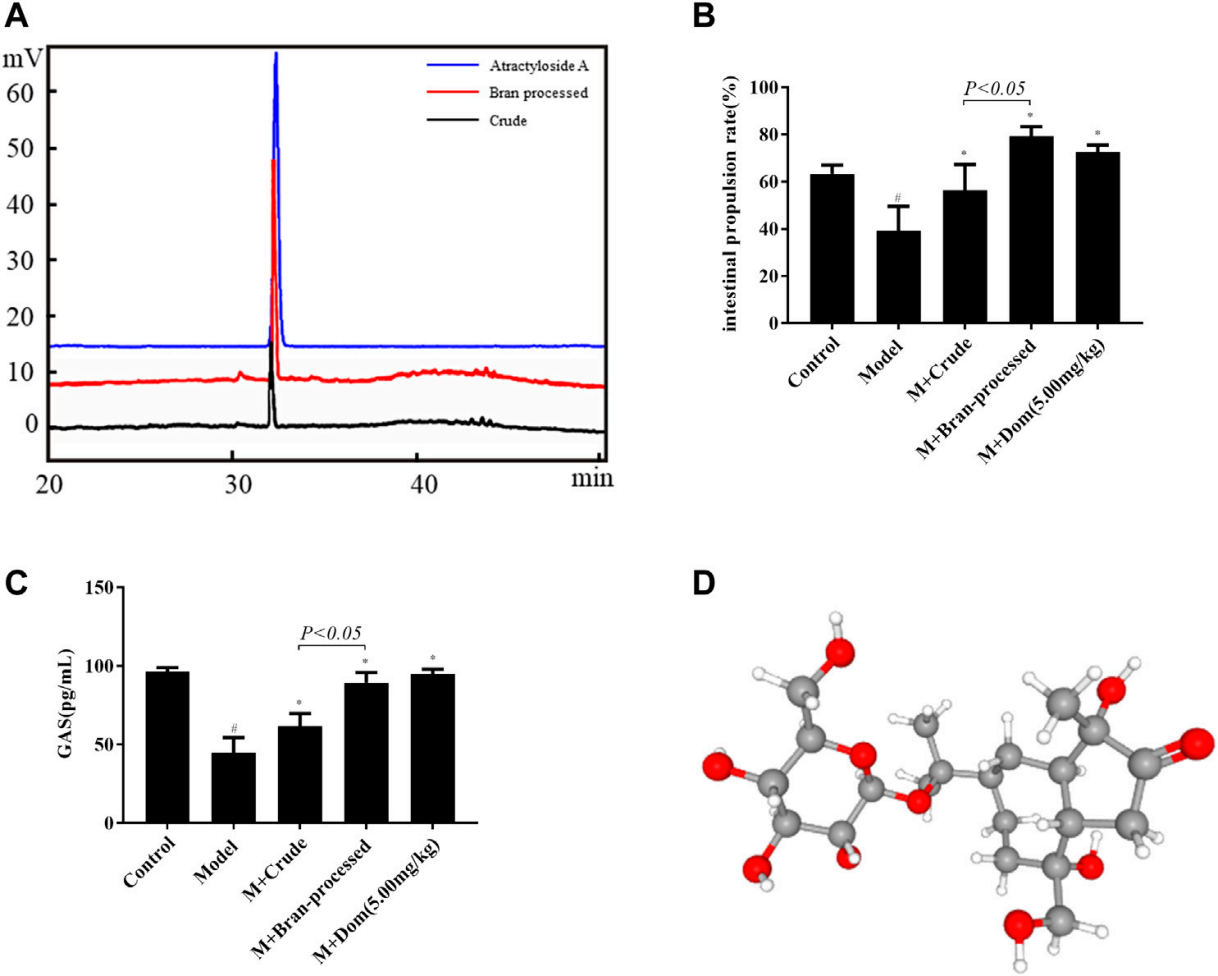
Identification of AA as the active ingredient in crude and bran-processed *Atractylodes lancea*. Model: SDS model rats group; M + Crude: SDS model rats administrated with crude AL extraction; M + Bran-processed: SDS model rats administrated with BAL extraction. **(A)** HPLC-ELSD chromatogram of the 50% EtOH extraction from AL (black) and BAL (red), the content of AA increased by 191.22% in the bran-processed *Atractylodes lancea*. **(B)** and **(C)** Comparison of the intestinal propulsion rate and GAS in SDS rats treated with crude and bran-processed *Atractylodes lancea* extracts. **(D)** 3D structure of AA. Data represented mean ± SD. Results are expressed as the mean ± SD from at least three independent experiments. *p < 0.05, compared with model; #*p* < 0.05, compared with control.

In TCM theory, BAL is considered more effective than AL for the treatment of SDS. In order to clarify the changes in pharmacodynamics between nonprocessed and bran-processed AL, AA was isolated and characterized using systematic solvent separation and HPLC-ELSD. As shown in Figure 1A, HPLC-ELSD analysis revealed that the concentration of AA increased by 191.22% in the bran-processed H_2_O-EtOH (50:50) fraction in comparison with crude AL, measured against a standard. Furthermore, following characterization of the compound by HPLC-MS, chemical and physical analyses were conducted, as displayed in supporting Information Supplementary Table S1, from which AA was finally identified as the compound of interest, after combining all the analysis data and published literature (Kitajima et al., 2003). The chemical and physical properties of AA are detailed in Supplementary Table S2. In addition, as presented in Figures 1B,C, bran-processed *Atractylodes lancea* was more effective in reversing SDS in the rat model, as it increased the intestinal propulsion rate more than crude AL. Based on previous studies, the results indicate that AA could be a key component involved in the spleen-invigorating effects of AL.

### AA Attenuated Spleen-Deficiency Syndrome-Induced Changes in Δbody Weight and Gastric Residual Rate, Increased Intestinal Propulsion Rate, and Increased the Levels of Gastrointestinal Motility Hormones

**FIGURE 2:**
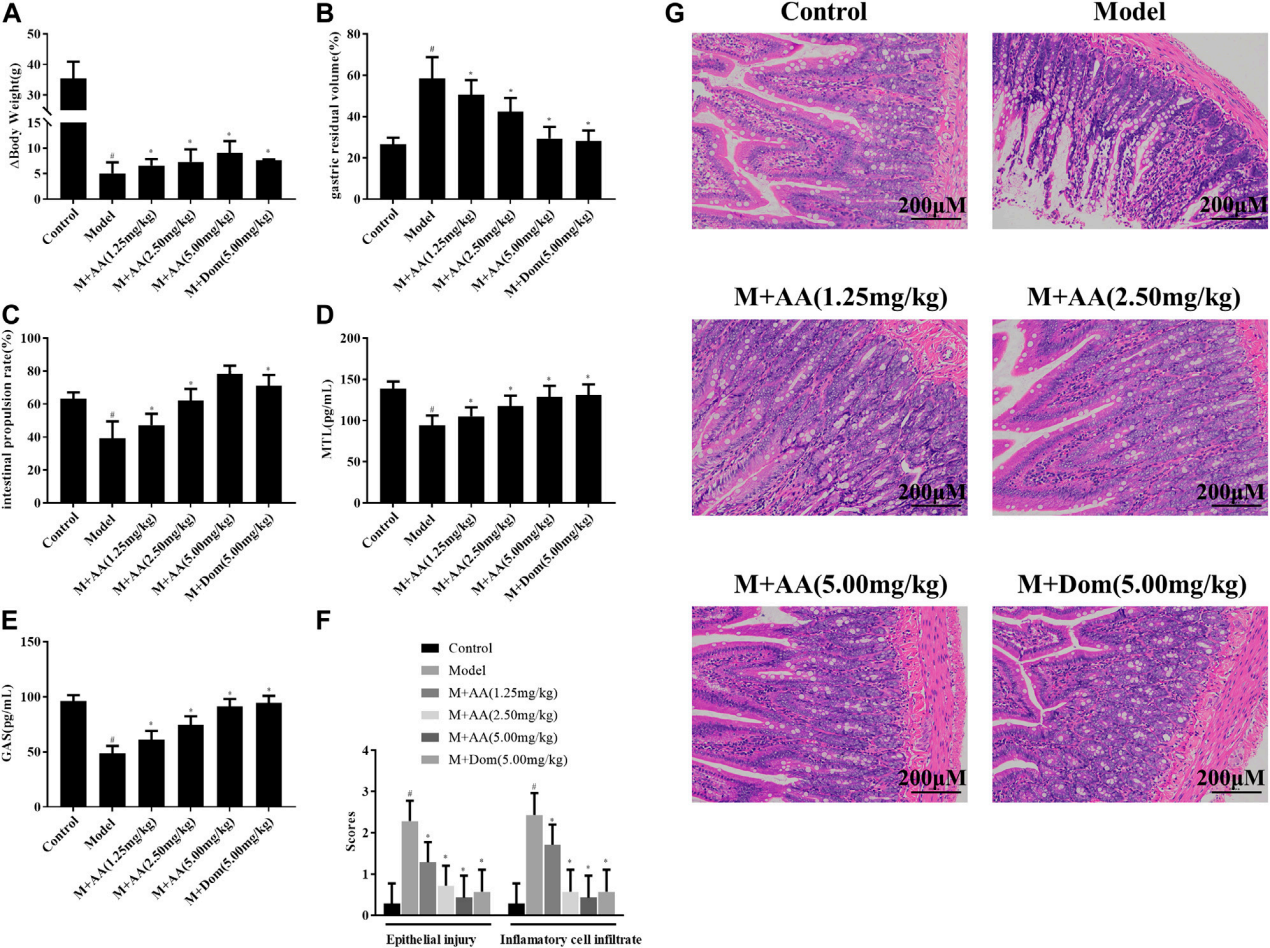
Effects of AA on Δbody weight, gastric residual volume, intestinal propulsion rate, and levels of gastrointestinal motility hormones in spleen deficiency syndrome rats (*n* = 10). Histopathological evaluation of the intestine of SDS rats treated with AA. M + AA: SDS model rats administrated with AA. **(A)** Δbody weight, **(B)** gastric residual volume, **(C)** intestinal propulsion rate, **(D)** serum gastrin levels, **(E)** motilin levels, **(F)** epithelial injury and inflammatory cell infiltration into intestine tissues of control, model, AA, and M + AA groups, *n* = 6 per group, and **(G)** representative images of HE-stained sections (200× magnification) showing pathological changes in SDS rats after treatment with low (1.25 mg/kg), middle (2.5 mg/kg), or high (5 mg/kg) doses of AA or with Dom (5 mg/kg). Data represented mean ± SD. Results are expressed as the mean ± SD from at least three independent experiments. *p < 0.05, compared with model; ^#^ *p* < 0.05, compared with control.

As shown in Figure 2A, the weights of rats in the SDS experimental model decreased significantly. After treatment with AA, their Δweights increased, a difference that was statistically significant with respect to the model group (*p* < 0.05). Besides, AA intervention affected the energy intake and appetite of SDS rats, as shown in Supplementary Figure S1. Furthermore, the gastric residual volume in the model group was higher than in the control group, and the intestinal propulsion rate was significantly lower in the model group compared with the control group (*p* < 0.05), as displayed in Figures 2B,C, suggesting that SDS caused gastrointestinal motility disorder in rats. After treatment with AA, gastric residual and intestinal propulsion rates returned to normal. Significant differences between the AA and Dom groups were observed compared with the model group (*p* < 0.05). As shown in Figures 2D,E, the levels of gastrointestinal motility hormones (GAS and MTL) were low due to spleen and stomach deficiency in the model group (*p* < 0.05), but after treatment with AA or Dom, the levels of MTL and GAS returned to normal and gastrointestinal function improved, with significant differences compared with the model group.

### Histopathological Evaluation of the Intestine of Spleen-Deficiency Syndrome Rats Treated With AA

SDS was induced by treating rats with a compound factor method, involving administration of an irregular diet, excessive fatigue, and exposure to a humid environment for 15 days. The degree of inflammatory cell infiltration into the intestine and epithelial injury was higher in the control and AA groups (*n* = 6) than in the model group (*n* = 6) (*p* < 0.05, Figure 2E). As shown in Figure 2G, histological analysis of the intestinal tissues indicated mucosal edema, inflammatory cell infiltration, and profound damage, including necrosis and shedding, in the SDS model group. After administration of AA, histopathological signs of intestinal injury decreased in a dose-dependent manner.

### AA Increased the C-Kit Expression Levels in the Intestine and Decreased PCNA and EGFR Expression Levels in the Gastric Tissues of Spleen-Deficiency Syndrome Rats

**FIGURE 3:**
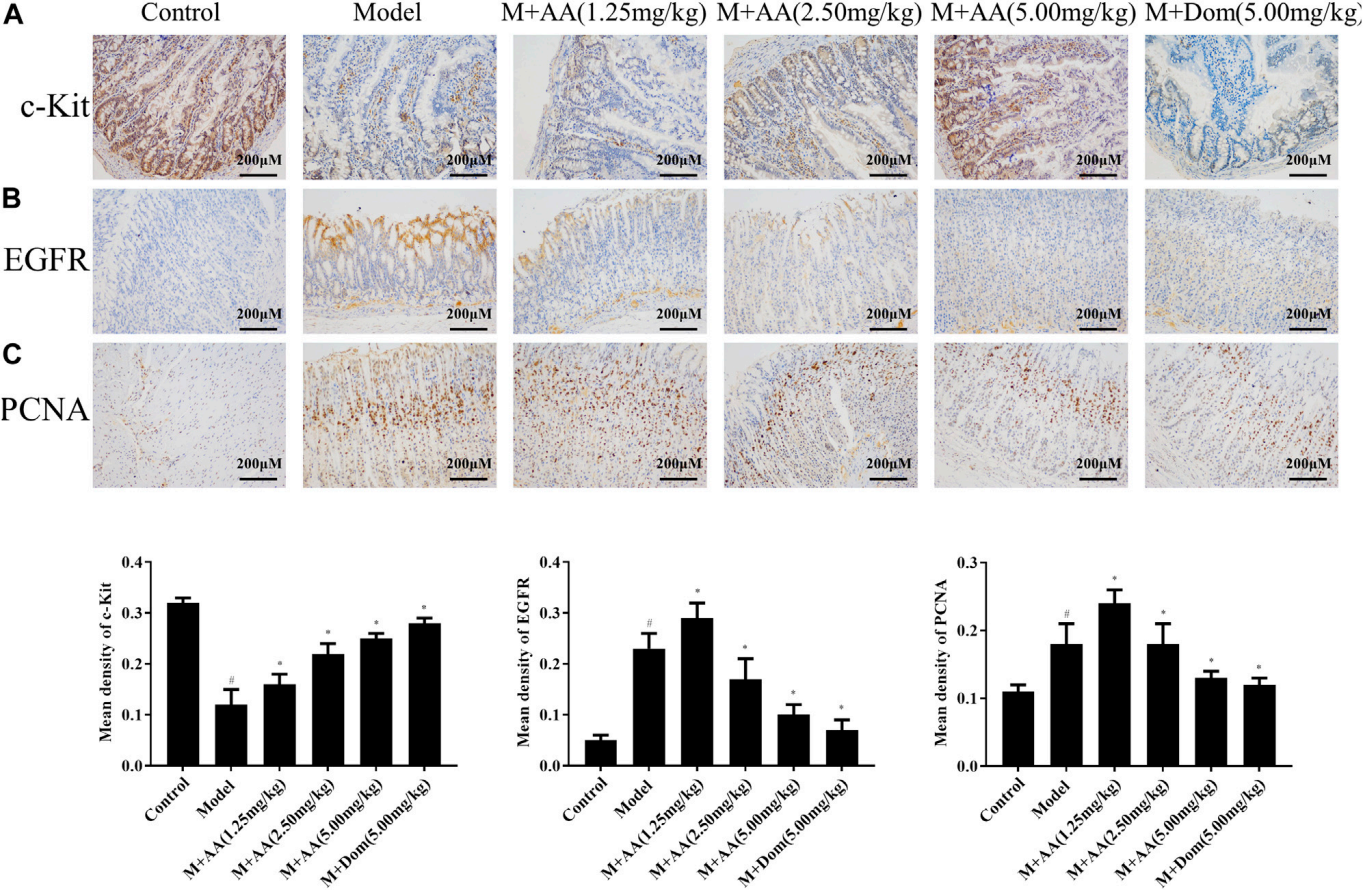
c-Kit protein expression in the intestine, and PCNA and EGFR protein expression in gastric tissues of SDS rats treated with AA (*n* = 10). Representative images of intestinal tissue sections (×200 magnification) stained with antibodies specific for **(A)** c-Kit, **(B)** EGFR, and **(C)** PCNA, showing the effects of AA at low (1.25 mg/kg), middle (2.5 mg/kg), and high (5 mg/kg) doses, as well as domperidone (5 mg/kg). Data represented mean ± SD. Results are expressed as the mean ± SD from at least three independent experiments. *p < 0.05, compared with model; ^#^ *p* < 0.05, compared with control.

As shown in Figure 3A, c-Kit expression was significantly reduced in the model group but increased significantly after treatment with AA or Dom (*p < 0.05*). Additionally, PCNA and EGFR protein levels increased significantly in the model group but decreased significantly after treatment with AA in a dose-dependent manner or after treatment with Dom (*p* < 0.05), as shown in Figures 3B,C.

### Potential Targets of AA and Functional Enrichment Analysis

**FIGURE 4:**
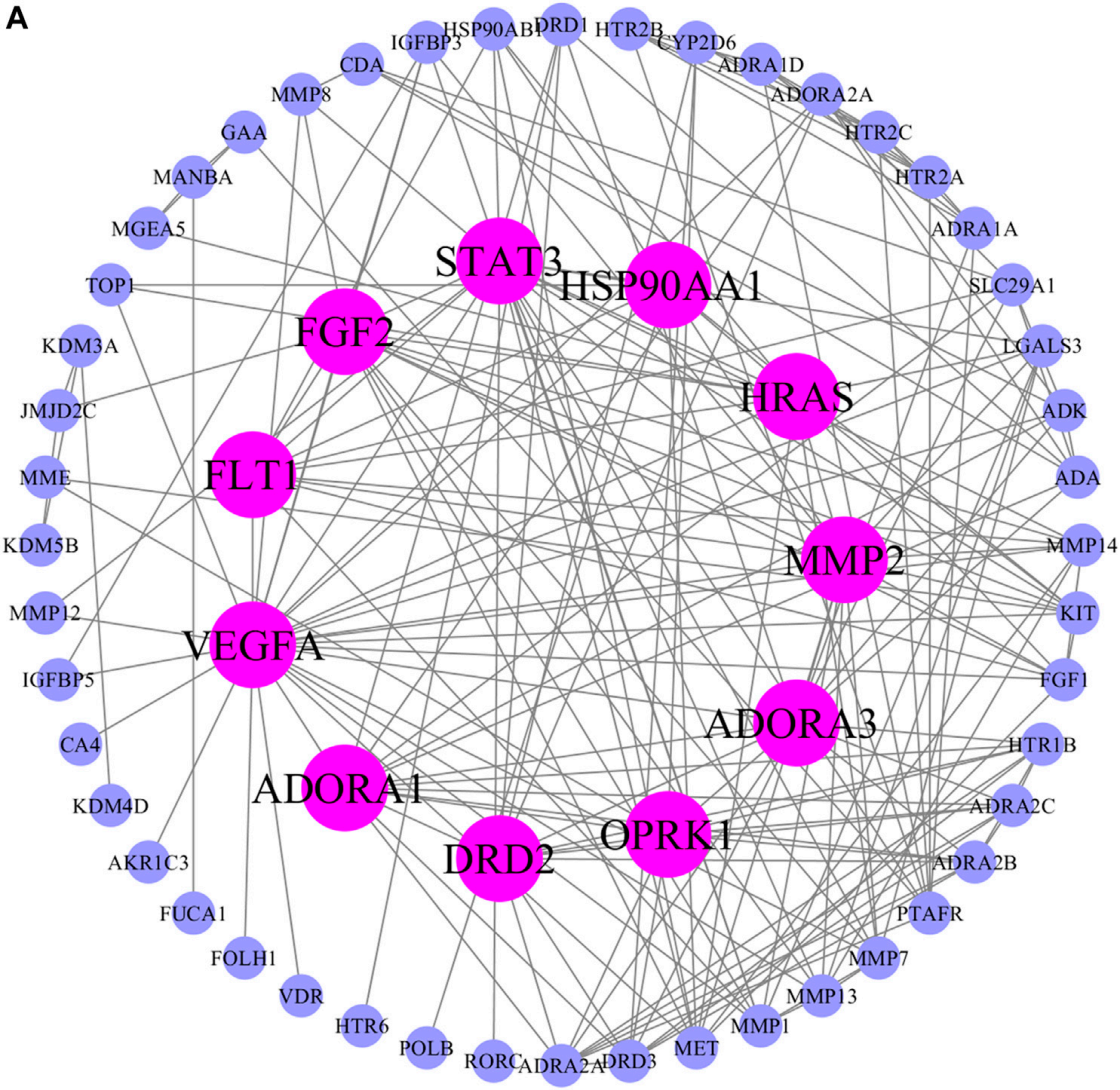
Protein-protein interaction network of potential AA targets. The pink nodes represent proteins with strong interaction potential. The blue nodes in the periphery represent proteins with weak interaction potential.

**FIGURE 5:**
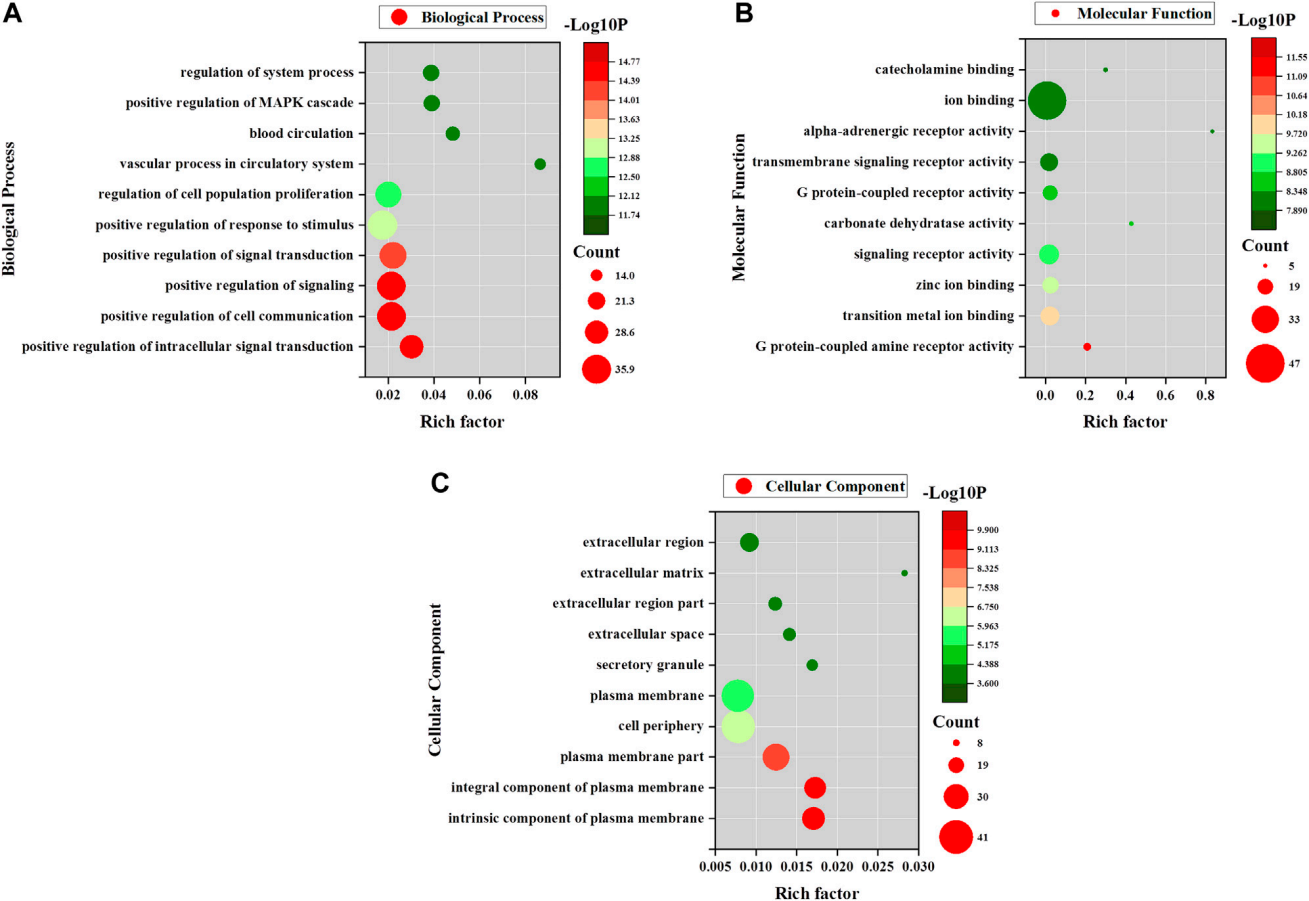
GO enrichment analyses for AA. **(A)** Biological process enrichment of potential AA targets. **(B)** Molecular function enrichment of potential AA targets. **(C)** Cellular component enrichment of potential AA targets.

**FIGURE 6:**
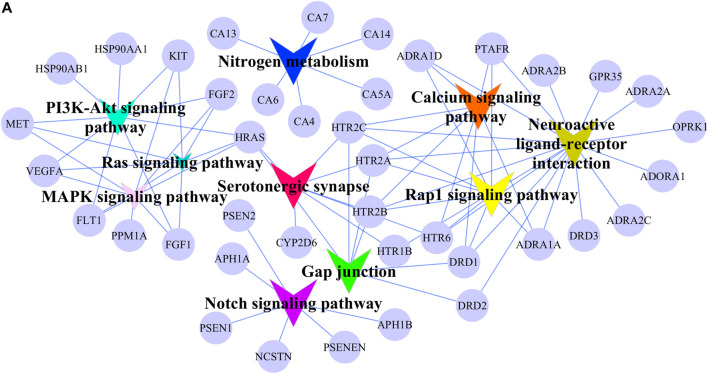
Signaling pathway network of potential AA targets. The circular nodes represent potential AA targets. The V nodes represent signaling pathways and different colors represent different pathways.

The possible pharmacologic mechanisms of action of AA were then explored. Using the Swiss Target Prediction database, 71 potential protein targets were identified, including FGF1, FGF2, FLT1, HRAS, KIT, MET, PPM1A, and VEGFA, as shown in Supplementary Table S3. Protein-protein interactions were identified, and GO functional and pathway enrichment analyses were conducted to establish relevant interactions, functions, and pathways of the 71 potential targets shown in Figure 4A. Functional analysis revealed that these putative targets mainly modulated signal transduction, including the MAPK cascade, as shown in Figures 5A–C. Pathway enrichment indicated that MAPK signaling was a principal pathway that was enriched, a pathway associated with gastrointestinal function, as shown in Figure 6A. The results suggest that AA suppresses the progression of SDS by targeting the MAPK signaling pathway.

### AA Directly Interacted With p38

**FIGURE 7:**
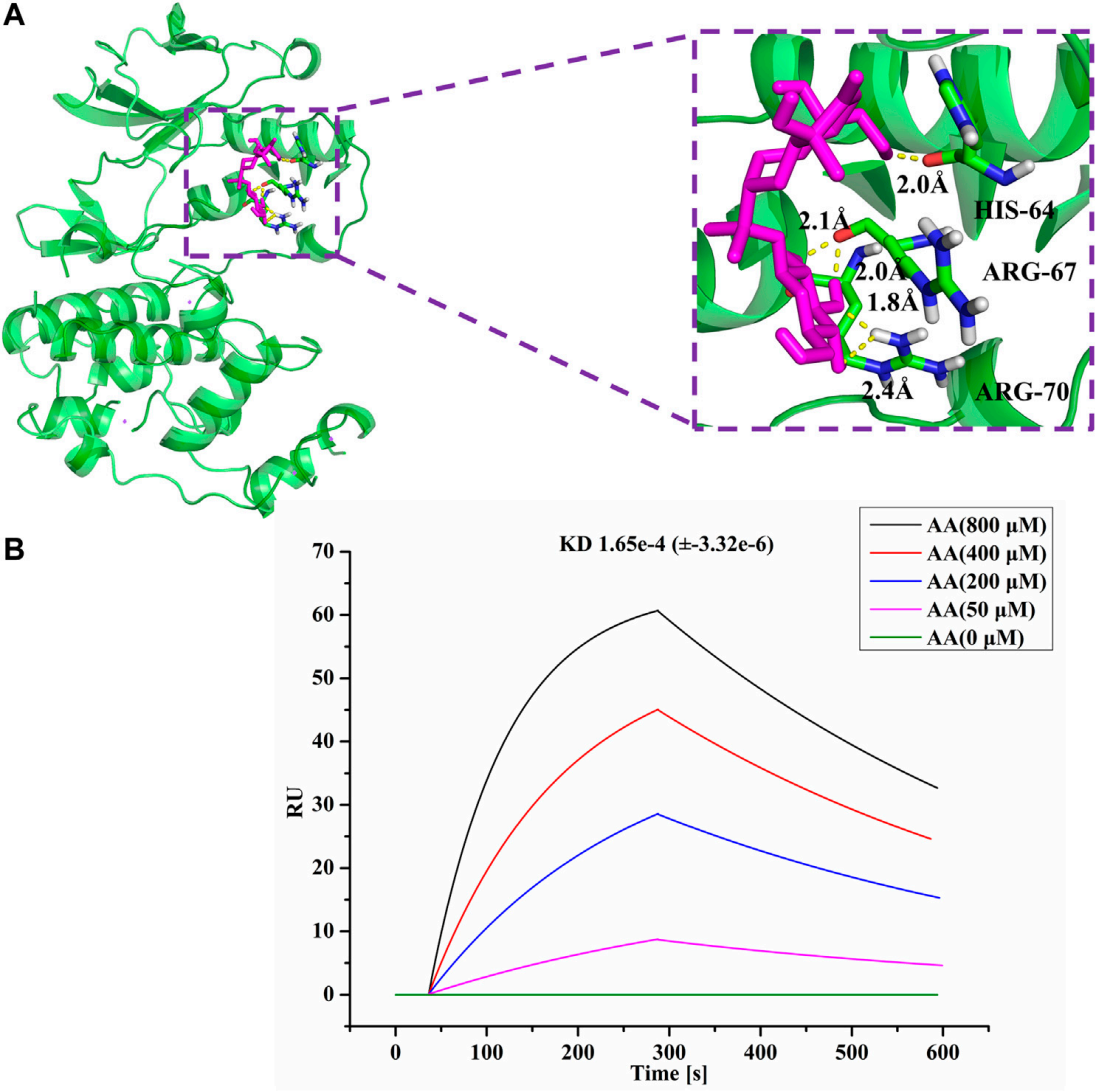
AA directly interacted with p38. **(A)** Molecules are represented by a ball-and-stick model, the hydrogen bonds are represented by a dotted line, and the distance is in angstroms. Atoms O and H of P38 are red and gray, respectively. **(B)** SPR analysis of the interaction between AA and p38.

As is well known, p38 of the MAPK signaling pathway is closely associated with an intestinal mucosal barrier function. Thus, we hypothesized that p38 may be associated with the function of AA in treating SDS. Hence, we conducted molecular docking of AA with p38, as shown in Figure 7A. AA docked to the predicted 3D model of p38 with a negative docking energy value (−4.42 kcal/mol). It was also apparent that the O atoms in AA and arginine (ARG)-70 (1.8 Å) and ARG-70 (2.4 Å) interact. The H atoms in AA interact with O atoms in histidine (HIS)-64 (2.0 Å) and ARG-67 (2.1 Å), respectively, in p38. Furthermore, using the SPR assay showed that AA directly bound to p38 (KD = 1.65e-4), as shown in Figure 7B. The results indicated that AA can suppress the progression of SDS by directly targeting p38, so as to inhibit the activity of p38 in the treatment of SDS.

### AA Modulation of Intestinal Mucosal Barrier Function through the MAPK Signaling Pathway

**FIGURE 8:**
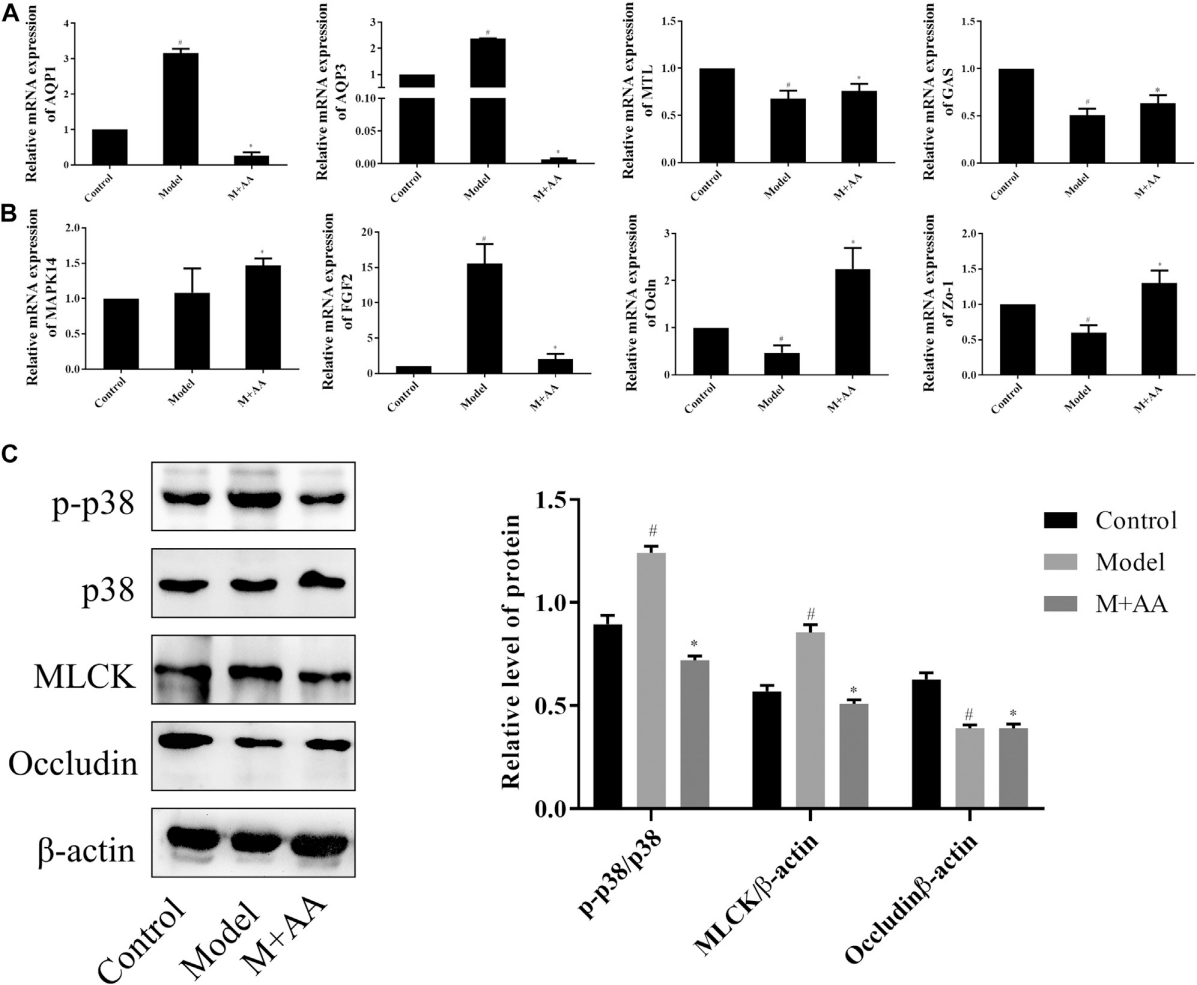
Effects of AA on signaling molecules that regulate intestinal mucosal barrier function in SDS rats (*n* = 10). SDS rats were treated with 5 mg/kg of AA. **(A)** and **(B)** Relative mRNA expression of intestinal metabolism-related genes determined by qRT-PCR. β-actin was used as the internal reference. **(C)** Protein expression levels determined by Western blot. Data represented mean ± SD. Results are expressed as the mean ± SD from at least three independent experiments. *p < 0.05, compared with model; ^#^ *p* < 0.05 compared with control.

Based on preliminary data analysis, the modulation of the intestinal mucosal barrier function by AA was further explored. To evaluate the activation of these signaling cascades, we first measured mRNA expression levels of genes associated with gastrointestinal function, in addition to the p38 MAPK signaling pathway. The results indicate that treatment with AA greatly increased MTL, GAS, ZO-1, and OCLN mRNA expression levels, while decreasing AQP1 and AQP3 mRNA levels, reported to be downstream of the MAPK pathway (Zhang et al., 2010; Zhao et al., 2017). FGF2 expression also declined. Interestingly, MAPK14 mRNA expression levels did not decline, suggesting that AA had no effects on p38 mRNA, as shown in Figures 8A,B. p38 phosphorylation, in addition to MLCK and occludin expression, was assessed by Western blot analysis. As predicted, levels of p38 phosphorylation and protein expression levels of MLCK increased significantly in the intestines of model rats compared with controls, whereas occludin protein expression decreased significantly with respect to controls. Remarkably, the administration of AA reversed these outcomes, as shown in Figure 8C. Collectively, the results indicated that AA can attenuate intestinal mucosal barrier dysfunction in rats with SDS, at least partly through the p38 MAPK signaling pathway.

## Discussion

Spleen deficiency is a major syndrome in TCM (Han et al., 2017) and intestinal mucosal barrier damage is considered among its main manifestations. It has been demonstrated that the intestinal mucosal barrier is the first line of defense against a hostile environment within the intestinal lumen, including pathogenic microorganisms. In addition, it plays important roles in metabolic regulation and maintaining the balance between the internal and external environments (Chen et al., 2019). In this study, we explored the protective effects of AA against intestinal mucosal barrier damage in SDS. It was designed from the basis of the traditional use of AL (Koonrungsesomboon et al., 2014). Recently, we reported that AL protects against spleen-deficiency-induced diarrhea (Shi et al., 2019). However, the active compound and the mechanisms underlying the therapeutic effects of this rhizome have not been fully elucidated. Glucosides are represented extensively in many TCMs, with versatile pharmacological and biological activities (Furman, 2015; Roh et al., 2020). Furthermore, the structure of the compounds in TCM is also closely related to their activity (Chen et al., 2018a; Chen et al., 2020). In the present study, AA was found to be a guaiane-type sesquiterpenoid glucoside from AL, which has three isoprenes and one β-D-glucopyranosyl unit. Through the use of a rat model of SDS and network pharmacology, we identified AA in an alcohol fraction of this herb, demonstrating that it improved intestinal mucosal barrier function by inhibition of p38-MAPK signaling pathway activation.

TCM theory emphasizes that spleen deficiency is a complicated pathological condition related to an imbalance of gastrointestinal function, resulting in weight loss, weak intestinal peristalsis, and inhibition of MTL/GAS secretion (Cai et al., 2011; Wang et al., 2012; Zhang et al., 2019). Associated symptoms include visceral hypersensitivity, high fecal water content, anxiety, depression, and slight colitis. In this report, we established a rat model of SDS based on the theory of traditional Chinese medicine. This was achieved by stimulation with a high fat/high sugar diet (lard and honey), a humid environment, and swimming-induced fatigue. Collectively, these symptoms coincided with those of spleen deficiency in TCM. The results demonstrated that treatment with AA increased Δbody weight, energy intake, and intestinal propulsion rate in SDS rats, while simultaneously decreasing gastric residual volume. As expected, MTL and GAS levels also increased. These results prove that AA provided a therapeutic effect. Moreover, H&E staining of the small intestine and IHC staining for c-Kit, EGFR, and PCNA further demonstrate that AA exhibits a considerable effect on gastrointestinal function.

Through a network pharmacology approach, we discovered more than 70 potential AA targets. Analysis of protein-protein interactions, GO function, and KEGG pathways indicated that the targets were enriched in several important signaling pathways, especially the MAPK signaling pathway. Furthermore, the results of docking and molecular interaction analysis indicated that AA suppressed the progression of SDS by directly targeting p38, thus inhibiting its activity in the treatment of SDS.

The MAPK pathway, a highly conserved signaling pathway, is involved in a variety of processes, including cell proliferation, survival, differentiation, and migration (Karin, 1998). There are four known MAPK pathways: ERK1 and 2; JNK/stress-activated protein kinase; p38 MAPK; and big mitogen-activated protein kinase (Sun et al., 2015). Previous studies have shown that p38 MAPK is involved in the negative regulation of tight junction proteins, which are important components of the intestinal barrier (Corre et al., 2017; Uwada et al., 2017; Liu et al., 2018). For example, the activated p38 MAPK pathway inhibits occludin expression by upregulating MLCK levels (Li et al., 2017; Al-Sadi et al., 2019). In the present study, we found that AA directly bound to p38 and downregulated p-p38 expression in addition to MLCK. Additionally, treatment with AA significantly increased occludin levels. The results also showed a decrease in AQP1 and AQP3 expression, which are downstream of the MAPK pathway. These results demonstrated that AA improves gastrointestinal function through the p38 MAPK pathway, at least in part.

## Conclusion

In summary, a comprehensive approach was undertaken to explore the mechanisms by which AA exerts a protective effect on the intestinal mucosa of SDS rats. The predictive capacity of this approach was supported by experimental evidence. Further studies of this kind may ultimately provide a deeper understanding of the molecular mechanisms underlying the therapeutic effects of AA on the spleen-deficiency syndrome.

## Data Availability Statement

All datasets presented in this study are included in the article/Supplementary Material.

## Ethics Statement

The animal study was reviewed and approved by Animal Ethics Committee of Hubei University of Traditional Chinese Medicine (Approval Number: 00273280).

## Author Contributions

JT, ZZ, and YL conceived and designed this study; JT, YX, and KX conducted the experiments; JT, DY, GC, LQ, and XL analyzed the data; JT, ZZ, and YL prepared the manuscript; JT, YX, and ZZ edited the manuscript. All authors approved the final version of the manuscript.

## Conflict of Interest

The authors declare that the research was conducted in the absence of any commercial or financial relationships that could be construed as a potential conflict of interest.
