# Supplementary material for: Treatment of Spleen-Deficiency Syndrome With Atractyloside A From Bran-Processed Atractylodes lancea by Protection of the Intestinal Mucosal Barrier
Source: Front Pharmacol. 2020 Nov 20;11:583160. doi: 10.3389/fphar.2020.583160 (PMC7919195; doi:10.3389/fphar.2020.583160)
Supplement: Supplementary file 1 [file Table1_v1.DOCX]

**Supporting Information**

**1. The method of isolation and purification of Atractyloside A**

The obtained n-butanol fraction (200 g) from AL was then subjected to large-aperture absorptive resin of D101 (20-60 mesh) and eluted with H_2_O-EtOH (100: 0, 50: 50, 5: 95) to obtain 3 fractions [(fr.A: H_2_O-EtOH (1:0), 168 g; fr.B: H_2_O-EtOH (1:1), 30 g; fr.C: H_2_O-EtOH (5:95), 1.5 g)]. Afterwards, the Fr.A was further separated to obtain 4 major fractions [Fr.A_1_ (12.15 g), Fr.A_2_ (1.24 g), Fr.A_3_ (10.20 g), Fr.A_4_ (1.34 g)] by silica column chromatography (100-200 mesh) with CHCl_3_-MeOH (100:0, 90:10, 80:20, 70:30, 30:70, 20:80, 10:90, 0:100, v/v) as eluate. Determined by HPLC-ELSD, the quantitative changing ingredient (Compound A) was found in FrA_3_, which was further chromatographed via silica column (200-300 mesh) using CHCl_3_-MeOH (20:80, 10:90, 0:100, v/v) as eluate. After analyzing by TLC profiles, the eluate was further combined and purified by Toyopearl (CHCl_3_-MeOH) to obtain the FrA_3-1_ (0.2 g). Finally, the FrA_3-1_ was further purified by Sephadex LH-20 column (MeOH) and preparative TLC to furnish the Compound A (21 mg). Then the Compound A was finally identified by a HPLC-MS which was performed on an Agilent 1100 system with a Varian INOVAS-600 MS.

**1.2 Chemical and physical analyses**

The compound is a white powder (mp 227- 230℃, [α]D+20.0 °) and its molecular weight is 448, easily soluble in aqueous EtOH and MeOH, but insoluble in petroleum, ethyl acetate and chloroform. It is non-fluorescent under UV, and the color reaction of Herb aldose-oil vitriol of TLC is red. Moreover, compound A shows a cluster ion peak [M+H] + at 447 in the positive fast atom bombardment mass spectrometry. The data of 13C-NMR (125MHz ,DMSO-d6)and 1H-NMR (600MHz, DMSO-d6) were showed in Supplementary Table S1.Above all, Compound A was finally identified as AA by combining with all the analysis data and published literature ([Shoji YAHARA et al. 1989](#_ENREF_29)) and its structure is shown in Figure 1.

**Supplementary Table S1. Data of 13C-NMR (125MHz, DMSO-d6)and 1H-NMR (600MHz, DMSO-d6)**

| Position | δ_H_ | Position | δ_c_ |
| --- | --- | --- | --- |
| H-1 | 2.83(1H,d,J=7.8) | C-1 | 41.65 |
| H-2 | 3.00(2H,d,J=5.4) | C-2 | 34.86 |
| H-5 | 2.46(1H,brdd,J=4.8) | C-3 | 219.72 |
| H-6 | 1.78(2H,dd,J=9.6) | C-4 | 76.99 |
| H-7 | 1.95 (1H,brddd,J=10.8) | C-5 | 44.62 |
| H-8 | 1.36(2H,brddd,J=9.0) | C-6 | 28.48 |
| H-9 | 2.23(2H,dd,J=5.0) | C-7 | 47.00 |
| H-12 | 1.06(3H,s,J=12) | C-8 | 21.28 |
| H-13 | 1.08(3H,s,J=13) | C-9 | 34.75 |
| H-14 | 3.35(2H,brs,J=11) | C-10 | 72.87 |
| H-15 | 0.89(3H,s,J=9.6) | C-11 | 79.85 |
| Glc H-1 | 4.24(1H,d,J=7.8) | C-12 | 23.15 |
|  |  | C-13 | 24.24 |
|  |  | C-14 | 68.76 |
|  |  | C-15 | 18.11 |
|  |  | C-1' | 96.90 |
|  |  | C-2' | 79.82 |
|  |  | C-3' | 76.53 |
|  |  | C-4' | 70.18 |
|  |  | C-5' | 73.59 |
|  |  | C-6' | 61.09 |

**Supplementary Table S2. The chemical and physical properties of AA**

| Property Name | Property Value |
| --- | --- |
| Molecular Weight | 448.5 g/mol |
| XLogP3-AA | -2.2 |
| Hydrogen Bond Donor Count | 7 |
| Hydrogen Bond Acceptor Count | 10 |
| RotaSupplementary Table Bond Count | 5 |
| Exact Mass | 448.230847 g/mol |
| Monoisotopic Mass | 448.230847 g/mol |
| Complexity | 671 |

**Supplementary Table S3. The potential targets of Atractyloside A**

| Target | Common name | Uniprot ID | ChEMBL ID | Target Class | Probability* |
| --- | --- | --- | --- | --- | --- |
| Nuclear receptor ROR-gamma | RORC | P51449 | CHEMBL1741186 | Nuclear receptor | 0.134972 |
| Gamma-secretase | PSEN2 PSENEN NCSTN APH1A PSEN1 APH1B | P49810 Q9NZ42 Q92542 Q96BI3 P49768 Q8WW43 | CHEMBL2094135 | Protease | 0.118883 |
| Heat shock protein HSP 90-alpha | HSP90ATRACTYLOSIDE A1 | P07900 | CHEMBL3880 | Other cytosolic protein | 0.118883 |
| Alpha-2a adrenergic receptor | ADRA2A | P08913 | CHEMBL1867 | Family A G protein-coupled receptor | 0.118883 |
| Adrenergic receptor alpha-2 | ADRA2C | P18825 | CHEMBL1916 | Family A G protein-coupled receptor | 0.118883 |
| Alpha-2b adrenergic receptor | ADRA2B | P18089 | CHEMBL1942 | Family A G protein-coupled receptor | 0.118883 |
| Alpha-1d adrenergic receptor | ADRA1D | P25100 | CHEMBL223 | Family A G protein-coupled receptor | 0.118883 |
| Dopamine D3 receptor | DRD3 | P35462 | CHEMBL234 | Family A G protein-coupled receptor | 0.118883 |
| Cytochrome P450 2D6 | CYP2D6 | P10635 | CHEMBL289 | Cytochrome P450 | 0.118883 |
| Serotonin 6 (5-HT6) receptor | HTR6 | P50406 | CHEMBL3371 | Family A G protein-coupled receptor | 0.118883 |
| Alpha-1a adrenergic receptor (by homology) | ADRA1A | P35348 | CHEMBL229 | Family A G protein-coupled receptor | 0.118883 |
| Serotonin 1b (5-HT1b) receptor (by homology) | HTR1B | P28222 | CHEMBL1898 | Family A G protein-coupled receptor | 0.118883 |
| Vitamin D receptor | VDR | P11473 | CHEMBL1977 | Nuclear receptor | 0.118883 |
| Serotonin 2b (5-HT2b) receptor | HTR2B | P41595 | CHEMBL1833 | Family A G protein-coupled receptor | 0.118883 |
| Vascular endothelial growth factor A | VEGFA | P15692 | CHEMBL1783 | Secreted protein | 0.118883 |
| Acidic fibroblast growth factor | FGF1 | P05230 | CHEMBL2120 | Secreted protein | 0.118883 |
| Basic fibroblast growth factor | FGF2 | P09038 | CHEMBL3107 | Secreted protein | 0.118883 |
| Platelet activating factor receptor (by homology) | PTAFR | P25105 | CHEMBL250 | Family A G protein-coupled receptor | 0.118883 |
| Serotonin 2a (5-HT2a) receptor | HTR2A | P28223 | CHEMBL224 | Family A G protein-coupled receptor | 0.118883 |
| Epoxide hydratase | EPHX2 | P34913 | CHEMBL2409 | Protease | 0.118883 |
| Sodium/glucose cotransporter 2 | SLC5A2 | P31639 | CHEMBL3884 | Electrochemical transporter | 0.118883 |
| Serotonin 2c (5-HT2c) receptor | HTR2C | P28335 | CHEMBL225 | Family A G protein-coupled receptor | 0.118883 |
| Adenosine A1 receptor | ADORA1 | P30542 | CHEMBL226 | Family A G protein-coupled receptor | 0.118883 |
| Adenosine A3 receptor | ADORA3 | P0DMS8 | CHEMBL256 | Family A G protein-coupled receptor | 0.118883 |
| Sodium/glucose-cotransporter 1 | SLC5A1 | P13866 | CHEMBL4979 | Electrochemical transporter | 0.118883 |
