# Supplementary figures and images for: Treatment of Spleen-Deficiency Syndrome With Atractyloside A From Bran-Processed Atractylodes lancea by Protection of the Intestinal Mucosal Barrier
Source: Front Pharmacol. 2020 Nov 20;11:583160. doi: 10.3389/fphar.2020.583160 (PMC7919195; doi:10.3389/fphar.2020.583160)

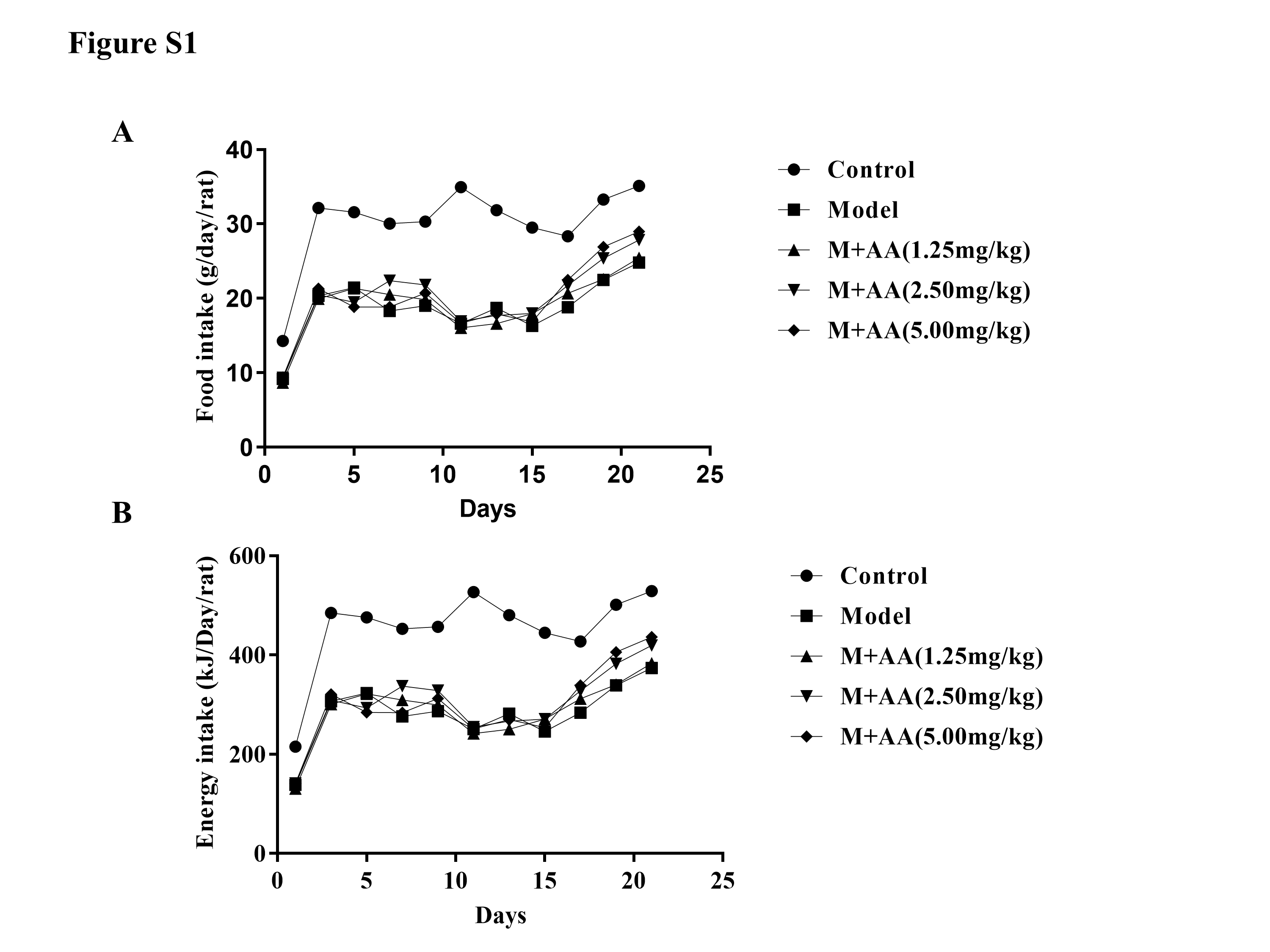

Supplement: Supplementary file 2 [file Image1_v1.TIF]
